# Implantation of Covered Stent for Coarctation of the Aorta and Secondary Hypertension in Adolescents—Case Report

**DOI:** 10.3390/children8111018

**Published:** 2021-11-05

**Authors:** Simina-Elena Ghiragosian-Rusu, Cristina Blesneac, Anca Sglimbea, Claudiu Ghiragosian, Laszlo Hadadi, Amalia Făgărășan, Rodica Togănel

**Affiliations:** 1 Department of Pediatrics III, George Emil Palade University of Medicine, Pharmacy, Science, and Technology of Targu Mures, 540142 Targu Mures, Romania; simina_r88@yahoo.com (S.-E.G.-R.); amalia_fagarasan@yahoo.com (A.F.); rodicatoganel@yahoo.com (R.T.); 2 Clinic of Pediatric Cardiology, Emergency Institute of Cardiovascular Diseases and Transplantation, 540136 Targu Mures, Romania; 3 Department of Interventional Cardiology, Emergency Institute for Cardiovascular Diseases and Transplantation, 540136 Targu Mures, Romania; aisglimbea@gmail.com (A.S.); hadadilaci@yahoo.com (L.H.); 4 Department of Surgery IV, George Emil Palade University of Medicine, Pharmacy, Science, and Technology of Targu Mures, 540142 Targu Mures, Romania; ghiragosian_claudiu@yahoo.com

**Keywords:** coarctation of the aorta, covered stent, adolescent, arterial hypertension, complication

## Abstract

Introduction: Coarctation of the aorta represents a narrowing of the thoracic aorta. Hypertensive patients with blood pressure differences ≥20 millimetres of mercury have an indication for surgical or interventional treatment. Implantation of a covered stent became the preferred therapy for the management of this pathology in adolescents/adults. Case report: We report the case of a 14-year-old male sportsman, who presented in the emergency room with headache, dizziness, and tinnitus. The clinical exam revealed blood pressure differences between the upper and lower limbs of up to 50 mmHg. Based on the clinical and paraclinical data, we established the diagnosis of coarctation of the aorta and severe secondary arterial hypertension. The case was discussed by a multidisciplinary team and accepted for covered stent implantation. The 24 h blood pressure Holter monitoring after the procedure indicated the persistence of stage I arterial hypertension. Conclusions: Coarctation of the aorta is a congenital cardiovascular anomaly with high morbidity and mortality rates. Arterial hypertension, heart failure, and aortic dissection are complications of this pathology, some of them being sometimes direct consequences of secondary hypertension. Periodic cardiology follow up after the procedure is mandatory to assess the hemodynamic response, to identify potential complications, and to stratify the cardiovascular risk.

## 1. Introduction

Coarctation of the aorta (CoA) represents a narrowing of the thoracic aorta, frequently localized at the insertion of the ductus arteriosus just distal from the left subclavian artery [1,2], with a prevalence of 4/10,000 live newborns [2], associated with other congenital cardiac disorders such as bicuspid aortic valve, aortic stenosis, ventricular septal defects, etc. [1]. It is more commonly seen in males (59% vs. 41%) [2].

The care of patients with this pathology depends on a series of factors such as the severity of the stenosis, age of the patient, and clinical manifestations. Both in children and in adults, delay in diagnosis can occur due to normal development and lack of symptoms [3,4,5]. The diagnosis is often incidental due to the presence of a systolic murmur and/or increased blood pressure values [2,3,4,5]. The presumptive CoA diagnosis can be based on the following clinical symptoms: weak/absent femoral pulse, systolic murmur, and blood pressure (BP) difference ≥20 mmHg between superior and inferior limbs. Moreover, the patient might complain of signs and symptoms related to arterial hypertension (AHT) such as headache, dizziness, epistaxis, or inferior-limb pains during physical effort [2,5]. Transthoracic echocardiography and thoracic computed tomography angiography (CTA) allow the accurate assessment of CoA anatomy [5,6,7,8].

The therapeutic approach in CoA is established by a multidisciplinary team. Options include surgery or percutaneous treatment [3]. Hypertensive patients with CoA and with BP differences ≥20 mmHg have an indication for surgical or interventional treatment [5]. The accurate assessment of the coarctation area and its surrounding anatomy is mandatory for establishing the type of coarctation [1,5,9,10]. The optimal management for coarctation of the aorta is controversial, and there is no comprehensive evidence-based standard of care or algorithm. For young children and infants presenting with native coarctation, the surgical repair is preferred by most centers [11]. The implantation of the covered stent became in time the preferred therapy for the management of this pathology in adolescents/adults, but it should not be expanded more than 10% as compared to the normal adjacent aorta [1,7,12]. The most common complications of this interventional treatment in both the short and long term are represented by residual arterial hypertension, vascular complications, restenosis, aortic aneurism, and the need for re-expansion once the patient grows (most frequently in teenagers) [13,14].

We present the following case report in order to underline the systemic implications of aortic coarctation in the life of adolescents and the techniques used to repair it and in order to contribute to early diagnosis in childhood since it is not customary to measure the pulse and blood pressure in both upper and lower limbs during physical examination.

## 2. Case Report

### 2.1. Presenting Symptoms

We report the case of a 14-year-old male sportsman (contact sport), without significant personal pathological history, who presented in the emergency room with the following symptoms: headache, dizziness, and tinnitus, and a clinical exam revealed elevated BP values (180/115 mmHg). It is worth mentioning that approximately 5 months ago elevated BP values were recorded at school but without any measures taken at that time.

### 2.2. Diagnostic Criteria

**Figure 1:**
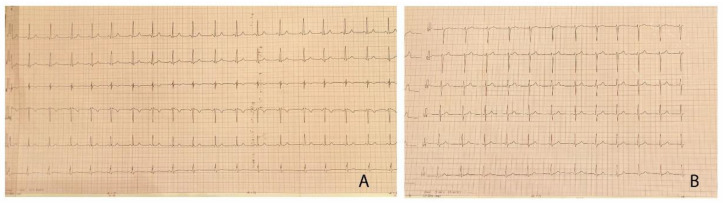
(**A**,**B**) 12-lead electrocardiogram of the patient, demonstrating left ventricular hypertrophy with Sokolow–Lyon index >35 mm and no signs of arrhythmia or conduction abnormalities.

The clinical exam at the time of admission revealed extreme anxiety, warm extremities, rhythmic heart sounds, grade III systolic murmur, with interscapulovertebral and bilateral carotid irradiation, the bilateral absence of femoral pulse, BP differences between the upper and lower limbs of up to 50 mmHg (right upper limb BP 180/115 mmHg, left lower limb BP 131/91 mmHg). We found no changes in the blood parameters, and the surface electrocardiogram pointed out left ventricular hypertrophy (LVH) (Figure 1). The 24 h BP Holter revealed BP values >95 percentile for age and height. The abdominal ultrasound showed no blood flow in the lobar and segmentary renal arteries.

**Figure 2:**
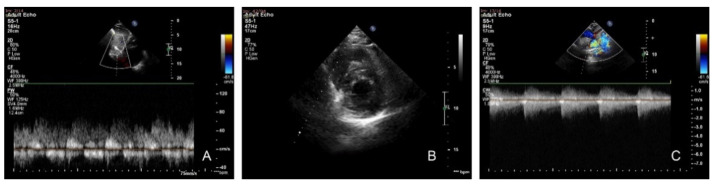
Transthoracic echocardiography. (**A**) Subcostal sagittal view: pulsed Doppler flow pattern in abdominal aorta—systolic waveform amplitude is low with the persistence of gradient in diastole. (**B**) Parasternal short axis: concentric left ventricular hypertrophy. (**C**) Suprasternal view: continuous-wave Doppler interrogation of the descending aorta. Note the high-velocity systolic amplitude (4.62 m/s) and maximum gradient of 85 mmHg with continuous antegrade flow through diastole.

Transthoracic echocardiography revealed a continuous curve on the Doppler interrogation of the abdominal aorta, concentric LVH, preserved systolic function of both ventricles, narrowing of the aortic isthmus, with a maximum gradient of 85 mmHg and diastolic run-off, and a severely dilated left subclavian artery (Figure 2).

**Figure 3:**
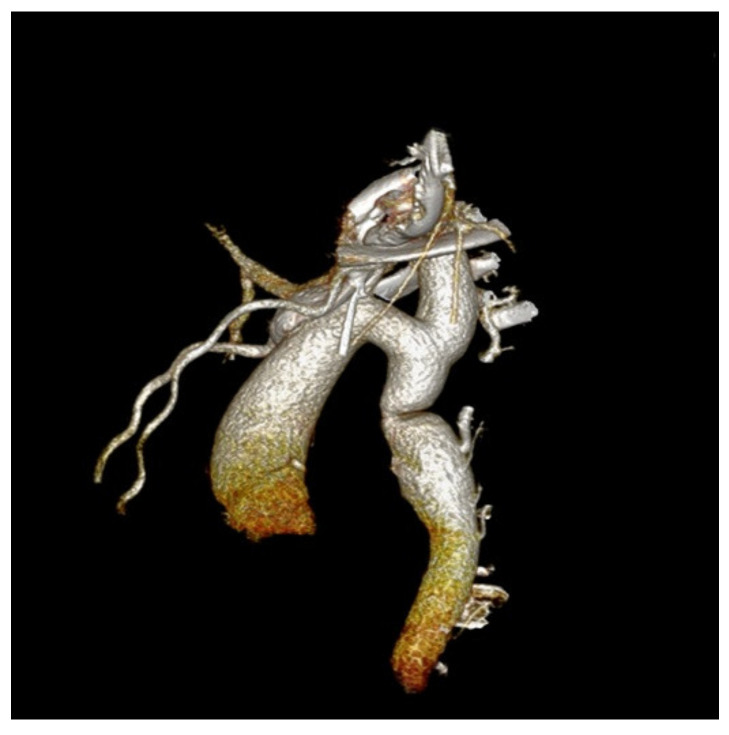
Angio-CT examination of the aorta precisely located the obstruction—a focal narrowing area, multiple collateral arteries, and bilateral dilated intercostal and subclavian arteries.

We performed a CTA in order to accurately assess the descending aorta, and we identified a focal narrowing of 0.7 cm diameter, multiple periscapular collateral arteries, and bilateral dilated intercostal and subclavian arteries (Figure 3).

Based on the clinical and paraclinical data, we established the diagnosis of isthmic CoA and severe secondary AHT.

### 2.3. Therapeutic Approach, Postprocedural Evolution, Cardiologic Follow Up

In terms of AHT, we decided to initiate antihypertensive drugs (beta-blockers) before the procedure, taking into account the result of the abdominal ultrasound, which were well tolerated hemodynamically.

**Figure 4:**
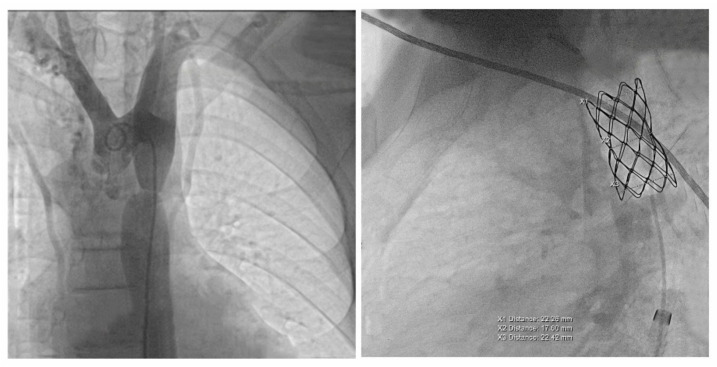
The first panel shows angio-fluoroscopic frames of the lesion in the antero-posterior projection, a localized narrowing at the isthmic level of 7 mm. The second panel shows the positioning of the 4.5 cm CP-covered stent.

**Figure 5:**
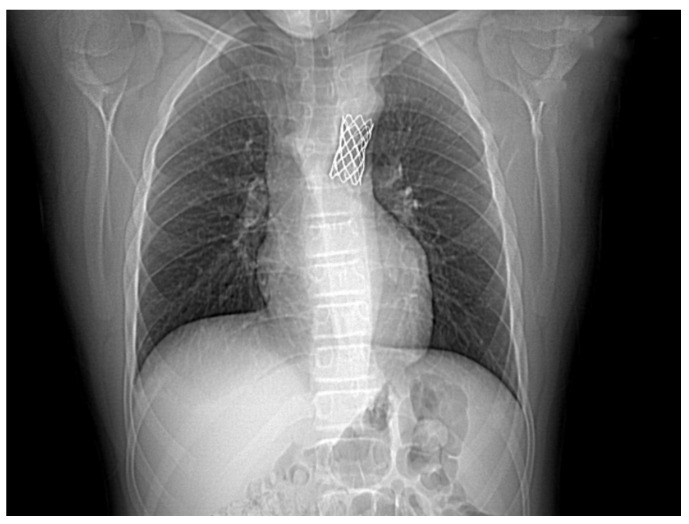
Control native chest CT visualizing the correct position of the stent.

The case was discussed by a multidisciplinary team and accepted for interventional treatment—implantation of covered stent under general anesthesia, with the patient ventilated with positive pressure using a laryngo-tracheal mask. The first contrast injection in the aortic arch was performed in the antero-posterior and latero-lateral projection, based on which, we identified a narrowing of the isthmic area of 7 mm, with a peak-to-peak gradient at this level of 23 mmHg, as well as a dilated left subclavian artery and multiple aorto-aortic collaterals. During the procedure, a Cheatham-platinum (CP)-covered stent of 4.5 cm was implanted on a balloon in balloon (BIB) of 20 mm × 5 cm inflated up to the burst pressure of 4 atm, which allowed the gradient reduction to 2 mmHg. The last three injections were performed in the antero-posterior and latero-lateral projection in the aortic arch, visualizing the normal position of the stent, without impairing the left subclavian artery’s emergence and without suggestive images for dissection or periaortic hematoma. These findings were also confirmed by the control native chest CT (Figure 4 and Figure 5).

The postprocedural echocardiographic assessment visualized the stent placed at the isthmic level with a maximum residual gradient of 22 mmHg and a mild persistence of the gradient in diastole along with an improvement in the pulsed color Doppler aspect of the abdominal aorta. On the 1st day after the procedure, the patient presented hypertensive episodes accompanied by severe anxiety resulting in the decision to adjust the dosage of the betablocker therapy and to associate an angiotensin-converting enzyme inhibitor, with subsequently controlled BP values.

#### Follow Up at 1 Month

The clinical exam pointed out bilaterally present femoral pulses, and no BP differences between the upper and lower limbs. The 24 h BP Holter monitoring indicated the persistence of stage I AHT, and therefore, we recommended the continuation of the antihypertensive therapy. The echocardiographic examination showed findings similar to those of the immediate postprocedural evaluation.

## 3. Discussion

CoA represents 4–6% of all congenital cardiac malformations [2]. Taking into account the increased mortality and morbidity of this malformation, an early diagnosis and treatment are essential. The commonest complications of CoA are represented by AHT, coronary disorders, sudden death, cardiac failure, stroke, endocarditis, aortic dissection, etc. [13,14,15]. The American Academy of Pediatrics recommends BP measurement in every clinically healthy patient over 3 years of age, in order to prevent diagnosis delays [5,16]. In our case, the patient was first detected with elevated BP at the age of 14 years, during a routine follow up at school, only 5 months prior to the admission and the diagnosis of CoA, without any other investigations or treatment measures taken during this time. It is worth mentioning that our patient was a performance sportsman.

The clinical diagnosis of CoA is based upon the characteristic findings of BP difference between the upper and lower limbs and diminished or delayed femoral pulses [2,5]. In our case, the absence of both femoral pulses was not observed until this age. According to data from the literature, the femoral pulse is furthermore diminished in the presence of multiple collaterals and the blood pressure difference between the upper and lower limbs is smaller [2,5]. In our case, the clinical exam revealed the absence of both femoral pulses, with BP differences of up to 40 mmHg despite the fact that the advanced imaging methods revealed several aorto-aortic collaterals.

Surgical repair has been the standard for this pathology, but in time the interventional treatment has replaced the surgical treatment in cases with appropriate anatomy. Regarding those cases presenting complex CoA anatomy, including those with transverse arch obstruction, tortuous segments of re-coarctation, distortion of adjacent arterial branches, or when repair of associated cardiac defects is needed, indication for surgical repair is suitable. Surgical repair of CoA can be performed by several techniques (resection with end-to-end anastomosis, subclavian flap aortoplasty, bypass graft across the area of coarctation of the aorta, etc.) [11,17].

The Coarctation of the Aorta Stent Trial (COAST) has been an influential prospective study examining the safety and effectiveness of interventional treatment in children and adults with CoA. Torok et al. suggested based-on literature data that the CP stent is a safe and effective treatment option for this pathology in older children and adults with native or recurrent CoA. Follow up for the COAST trial is planned for up to 60 months after stent positioning and will provide further insight regarding these issues [11]. The study dates, comparison between surgical and interventional treatment in the management of CoA indicates nearly equal effectiveness, the mortality rates are also similar and are more related to the associated cardiac defects, but surgery requires longer hospital stay and implies greater morbidity, higher costs, and higher complication rates [18].

The implantation of a covered stent in CoA patients became the preferred therapy, proving its efficacity in 96–98% of the cases [16]. It has recently become one of the most important and commonly used therapeutic approaches in severe cases of CoA, mainly due to the low risk of injuries to the aortic wall such as aneurisms/dissection [19,20]. The use of covered stents is preferred because of lower short- and long-term complication rates, as specified by Taggart et al. [21]. Nevertheless, injuries of the aortic wall might occur but with limited hemorrhage, excepting the cases when the sealing was insufficient or when the stent coverage was broken [20]. This procedure should be performed under general anesthesia due to the pain caused by stent implantation once the CoA is dilated. Similarly, for this procedure, our patient underwent general anesthesia with positive-pressure ventilation via a mask, with 30% O_2_ [21]. As in our case, retrograde access in CoA is the most common approach for interventional treatment [19,20,21]. The stent type, length, and diameter are established during the procedure, depending on the anatomy of the malformation and angiographic measurements. Moreover, the stent’s ability to be dilated for adult sizes and its position in relation to the surrounding vessels should be taken into consideration when choosing the stent. Once the stent type and diameter are established, the balloon and, in turn, the sheath used for the implantation should be carefully selected [21]. In the case reported above, a CP-covered stent of 4.5 cm was implanted and dilated with a BIB of 20 mm × 5 cm (up to a burst pressure of 4 atm), introduced via the right femoral artery, through a 14F sheath, without intra- or postprocedural incidents.

Stent implantation for CoA is considered a success when the pressure gradient measured during the procedure is <10 mmHg, detecting at the same time an improvement in the aortic lumen of >90% of the diameter of the normal adjacent aortic arch vessel [1]. Stassen et al. performed a retrospective study on 89 patients who benefited from covered stent implantation and described a significant reduction of the pressure gradient between the ascending and descending aorta from 25 ± 16 to 7 mmHg [3,20]. In our case, the angiographic measurements of the pressure in the ascending aorta and the femoral artery pointed out a peak-to-peak gradient of 23 mmHg and a postprocedural residual gradient of 2 mmHg, the procedure being, therefore, considered a success.

In terms of secondary and residual AHT, in the aforementioned study, the authors pointed out an improvement of BP values at three months after the procedure, without the normalization of the BP profile and with residual AHT being considered a frequent complication after CoA correction. According to this study, the patients remain exposed to an increased cardiovascular risk with premature morbidity and mortality [20]. Other studies proved that approximately 30% of the teenagers and 60% of the adults treated surgically/interventionally for CoA presented residual arterial hypertension [5,22,23]. Moreover, 24 h BP monitoring within three days from the procedure and after 1 month revealed in our patient the persistence of first-degree arterial hypertension. Regular cardiology follow ups with careful BP monitoring and low threshold for residual AHT diagnosis are important for establishing the long-term strategy, in order to stratify the cardiovascular risk for morbidity and mortality.

## 4. Conclusions

CoA is a common congenital cardiovascular anomaly with high morbidity and mortality rates, which is frequently misdiagnosed. AHT, coronary disorders, sudden death, heart failure, stroke, endocarditis, and aortic dissection are complications that stem from the evolution of this pathology when left untreated, some of them being sometimes direct consequences of secondary hypertension. Covered stent implantation in patients with CoA has become the preferred therapy for teenagers and adults due to the reduced risk of aortic wall injuries and proved efficiency in more than 95% of the cases. Periodic cardiology follow up after the procedure is mandatory in order to assess the hemodynamic response, to identify the potential complications, and to stratify the cardiovascular risk.
